# Cyclic neutropenia with a novel gene mutation presenting with a necrotizing soft tissue infection and severe sepsis: case report

**DOI:** 10.1186/s12887-015-0352-5

**Published:** 2015-04-02

**Authors:** Yoon Jung Boo, Myung Hyun Nam, Eun Hee Lee, Kuang Chul Lee

**Affiliations:** Division of Pediatric Surgery, Department of Surgery, Korea University College of Medicine, 73 Inchonro, Sungbuk-gu, Anam-dong, Seoul, 136-705 Korea; Department of Laboratory Medicine, Korea University College of Medicine, Seoul, Korea; Department of Pediatric, Korea University College of Medicine, Seoul, Korea

**Keywords:** Cyclic neutropenia, Soft tissue infection, Necrotizing, Negative-pressure wound therapy

## Abstract

**Background:**

Cyclic neutropenia is a rare disease. We report a 31-month-old girl with congenital cyclic neutropenia with a novel mutation in the *ELANE* gene who developed an acute necrotizing soft-tissue infection on her left axillary legion.

**Case presentation:**

A 31-month-old girl was admitted to our pediatric emergency room because of a necrotizing soft tissue infection of the left axillary area. The infection progressed rapidly and resulted in septic shock. Despite a medical treatment and surgical debridement, the sepsis was not controlled, and severe inflammation developed. After applying of negative-pressure wound therapy, her clinical symptoms improved. Finally, she was diagnosed with cyclic neutropenia with a novel genetic mutation. One month after admission, she was discharged with a completely recovered wound and no need for skin grafting.

**Conclusion:**

Both adequate medical treatment and effective control of the source of infection are critically important to reduce morbidity in such complex cases of necrotizing fasciitis as appeared in an immunocompromised pediatric patient.

## Background

Cyclic neutropenia (CN) is an uncommon hematologic disorder characterized by periodic neutropenia (<0.2 × 10^9^neutrophils/day for 3-5days) with an average 21-day turnover frequency [1]. Untreated children typically display fever, mouth ulcers and recurrent oropharyngeal infections. Cellulitis is common during neutropenic periods, but bacteremia and severe sepsis are rare [1]. Necrotizing fasciitis is also a rare event in the pediatric population. When it occurs, however, if not promptly diagnosed it can lead to severe wound problems that are difficult to manage. It can even lead to patient’s mortality. We report a 31-month-old girl with CN who developed acute necrotizing fasciitis in her left axillary region. Her diagnosis was confirmed by a mutational analysis of the *ELANE* gene, the causative gene of CN, which revealed a novel mutation. The infection progressed rapidly and resulted in septic shock. Treatment with negative-pressure wound therapy (NPWT) rescued the patient through effective control of the source of infection.

## Case presentation

A 31-month-old girl was admitted to our pediatric emergency room because of fever and a skin lesion in her the left axillary region. The patient’s mother suggested that she might have bitten by a mosquito 2 days previously. Her initial vital signs were stable: blood pressure 90/60 mmHg, heart rate 100 bpm, body temperature 37.6°C. She complained of pain in the axillary area, and her level of consciousness was alert. She appeared dehydrated and slightly subdued. A full blood count on admission demonstrated a total white blood cell count of 3,400/ μL and a neutrophil count of 329/ μL. Her medical history revealed that she had been hospitalized several times because of pharyngitis and bronchiolitis. Most of these admissions lasted fewer than 6 days. Unusual neutropenic events were noted during her previous hospitalizations, which raised the suspicion of CN. A pediatrician recommended a further evaluation to confirm this diagnosis, but her parents refused.

She was hospitalized, and both antibiotics (piperacilin plus tazobatam) and fluid therapy were commenced to treat the suspected cellulitis. Within 8 hours, however, she had deteriorated, requiring inotropic support and intubation for the management of respiratory failure. At that point, the patient was referred to a pediatric surgeon for a consultation regarding the axillary legion.

**Figure 1:**
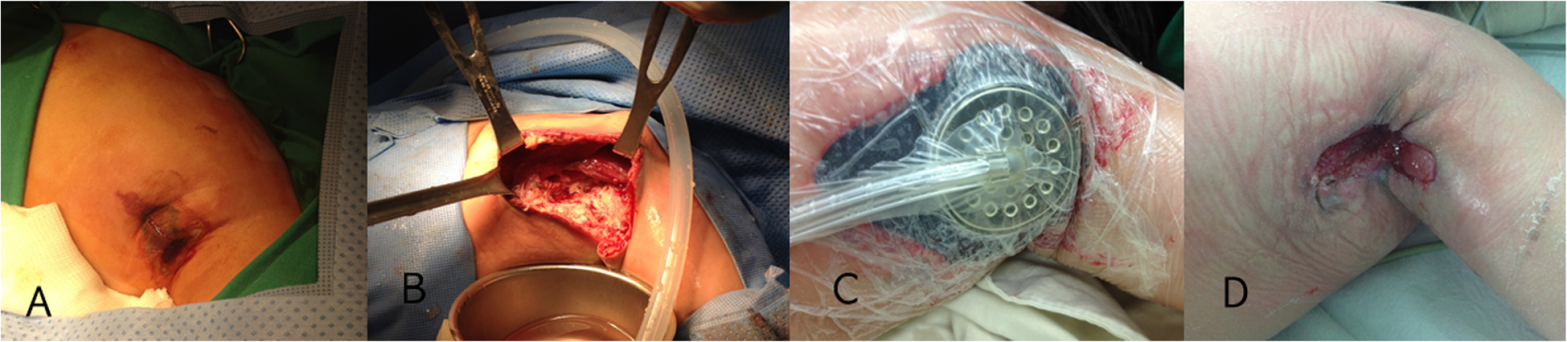
**Successful treatment of the axillary wound. (A)** The initial lesion on the axilla comprised swollen edematous skin with a bluish change at the center. **(B)** Note the massive necrosis of the subcutaneous area, muscles, and fascia with contaminated discharge at the initial surgery. **(C)** View after negative-pressure wound therapy. **(D)** After 1 month, granulation tissue grew rapidly to enable primary repair.

On physical examination, the lesion was swollen, edematous, and showed crepitant skin with a bluish area at the center (Figure 1A). With a diagnosis of necrotizing fasciitis, extensive surgical debridement was undertaken. During surgery, massive debridement of necrotic tissues and copious irrigation were performed, combined with the application of external drainage catheters (Figure 1B). *Pseudomonas aeruginosa* and *Staphylococcus hominis* were isolated from exudates of the wound, and *P. aeruginosa* was isolated from the blood as well. In the intensive care unit, she was managed with broad-spectrum antibiotics (cefepime with imipenem) and parenteral nutritional therapy. The isolated *Pseudomonas* was resistant to ampicillin and cephalosporin, but susceptible to imipenem. *Staphylococcus hominis* was resistant to beta-lactam antibiotics and susceptible to vancomycin. The antibiotics were changed to vancomycin with imipenem according to the antibiotics sensitivity report of these organisms.

**Figure 2:**
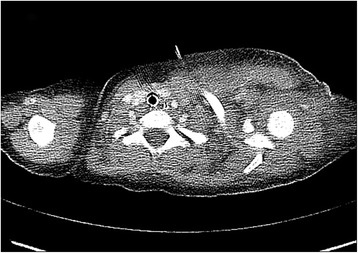
**Computed tomography shows diffuse swelling of the lateral chest wall and axilla 4 days after the initial surgery.**

Granulocyte-colony stimulating factor (G-CSF) was also administered because of the strong suspicion of underlying CN. The neutropenia normalized 2 days after the G-CSF treatment. She underwent daily surgical wound debridement and irrigation to control the source of infection. The sepsis was not controlled, moreover, the inflammation spread to her chest wall and neck area. Computed tomography 5 days after the initial surgery showed diffuse swelling of the left lateral chest wall and axilla, indicating further spread of the inflammation (Figure 2). Five days after the initial treatment, we applied negative-pressure wound therapy (V.A.C., KCI, San Antonio, TX, USA) after surgical debridement (Figure 1C). The negative pressure was gradually increased to 100 mmHg, and the wound dressing was changed every 3 days. Several days later, her clinical symptoms dramatically improved, and she was transferred to the general ward. Granulation tissue grew so rapidly that she did not require mesh grafting or skin grafting to repair the wound (Figure 1D). One month after admission, she was discharged with a completely recovered wound.

**Figure 3:**
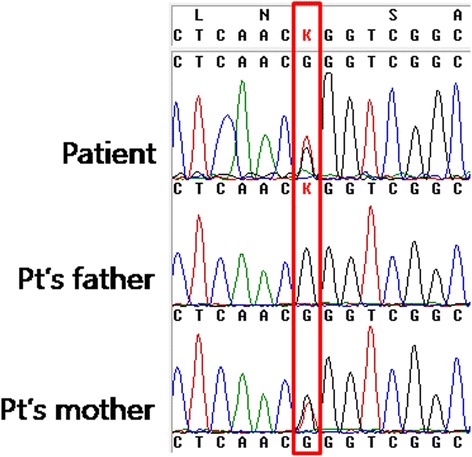
**DNA sequencing analysis of the** ***ELANE*** **gene.** Note that both the patient and her mother carry the same mutation (c.373G > T, p.Gly125Trp).

Considering her past history and clinical course, her underlying disease was suspected to be CN. Serial measurements of absolute neutrophil counts for hospitalizations, including laboratory data from the outpatients department, showed neutrophil count oscillations with a 21-day periodicity and neutropenia at the nadir of the cycle. We evaluated the patient to rule out other diseases that cause severe neutropenia. Laboratory tests for immunologic, hematologic and other genetic disorders were performed with no abnormal findings. Therefore, we recommended that her parents to take a family consultation for genetic analysis. The family history revealed that her mother had suffered from recurrent infections and was diagnosed with CN 4 years previously at another hospital. In addition, her grandmother died in her fourth decade as a result of a presumptive diagnosis of hematologic malignancy. The patient’s *ELANE* gene was analyzed by polymerase chain reaction analysis and direct sequencing. A heterozygous sequence variation was noted in exon 4 of the *ELANE* gene at codon 125 (c.373G > T, p.Gly125Trp). After a genetic analysis, the same mutation was detected only in her mother (Figure 3). This variation was not found in the Human Gene Mutation Database (HGMD) [2].

## Discussion

To our knowledge, this is the first reported case of septic shock caused by a necrotizing soft-tissue infection occurring in a patient with CN. CN is an uncommon disease characterized by cyclic episodes of severe neutropenia occurring typically at 21-day intervals and resulting in frequent opportunistic infections [3]. During these periods of neutropenia, patients may suffer painful mouth ulcers, gingivitis, lymphadenopathy, fever, pharyngitis/tonsillitis, and bacterial infections. Skin infections such as cellulitis are also common. In contrast to severe CN, however, serious infection and sepsis are rare [4]. Necrotizing soft-tissue infection is also a rare disease that can quickly progress to a life-threatening condition, with a greater risk in immunocompromised individuals. Prompt diagnosis and adequate treatment, including broad-spectrum antibiotics and surgical debridement are necessary to prevent morbidity and mortality. In cases of necrotizing soft-tissue infection, adequate surgical debridement is necessary to keep the infection from spreading. Most patients, even children, require repeated debridement [5]. Additional exploration, however, can lead to repetitive general anesthesia, which could be a great burden for pediatric patients.

Negative-pressure wound therapy is a recently introduced procedure that uses controlled negative pressure (vacuum) to help promote wound healing [6,7]. It enables the patient to avoid repetitive surgical debridement and general anesthetic procedures by effectively controlling the infected wound, as in our case. After the application of negative-pressure wound therapy, we could manage the patient’s wound with minimal doses of sedatives without the need for general anesthesia. Successful control of the source of infection helped the patient recover from her septic condition. Moreover, rapidly growing granulation tissue prevented her from having to undergoing further procedures (e.g., skin grafting) that are often required in cases of necrotizing soft-tissue infection.

*Pseudompnas aeruginosa* can cause of necrotizing soft-tissue infections that result in mortality. Necrotizing soft-tissue infections are categorized into three types. Type I is a mixed infection with aerobic and anaerobic bacteria (70-80%). Type II is a group Aβ-hemolytic *Streptococcus* spp. and *Staphylococcus aureus* (20-30%) mixed infection. Type III is Gram-negative monomicrobial infection [8]. *Staphylococcus aureus* and group A *Streptococcus* spp. are the predominant pathogens in the United States and Europe, whereas monomicrobial Gram-negative pathogens such as *Vibrio vulnificus*, *Klebsiella pneumoniae*, and *E.coli are common in Asia* [8]. A few cases of *Pseudomonas* necrotizing infection have been described, but most were of the eyelid infections [9]. The invasive mechanism of this unusual pathogen is unclear. We inferred that this organism might have originated from the colonization of her skin during a previous hospitalization. By compromising her local defenses, this bacterium could proliferate rapidly, causing the destructive infection that lead to septic shock.

CN is a rare autosomal dominantly inherited neutropenic disorder caused by a mutation of the *ELANE* gene located on chromosome locus 19p13.3, which encodes neutrophil elastase. Mutations in *ELANE* are found in nearly 100% of individuals with typical features of CN and in 38 - 80% of individuals with congenital neutropenia. The mutations were found to be distributed throughout the length of the gene, rendering any clear pattern (if one exists) less obvious [10]. *ELANE* mutations are thought to lead to a “gain-of-function” in the neutrophil granule protein leukocyte elastase (e.g., aberrant processing, packaging), causing cellular toxicity in neutrophil precursors. We performed further analysis to determine if this heterozygote sequence variation (p.G125W missense variant of ELANE gene) affects protein function. Because the sequence variations are not known as mutations, we performed two online virtual protein function prediction analyses to predict whether the amino acid substitution found might affect protein function. Using the PolyPhen analysis tool, the variation was predicted as “probably damaging” to protein function. Using the SIFT tool, the result was “Damaging” to protein function [11]. Therefore, the p.G125W missense variant of *ELANE* gene may negatively affect neutrophil elastase function, which plays a pathophysiologic role in CN [10].

## Conclusion

We report that a necrotizing soft-tissue infection with severe sepsis occurred in a patient with CN containing a novel mutation in the *ELANE* gene. In this immunocompromised pediatric patient, adequate medical treatments with antibiotics and G-CSF plus effective control of the source of infection were critically important to reduce morbidity. For suspected patients, genetic counseling and analysis should be performed for a better understanding of the genetic and molecular mechanisms of CN.

### Consent

Written informed consent was obtained from the patient’s legal guardian for publication of this Case report. A copy of the written consent is available for review by the Editor of this journal.

## Abbreviations

CN: Cyclic neutropenia
*ELANE*: elastase, neutrophil expressed
